# Silica Fume as a Physical Dispersing Agent for Carbon Nanotubes in Cementitious Mortars: Microstructural Mechanisms, Mechanical Performance and Carbon Reduction Efficiency

**DOI:** 10.3390/nano16140885

**Published:** 2026-07-18

**Authors:** Alaíde Marta dos Santos, Viviany Geraldo, Rovadávia Aline de Jesus Ribas, Wanna Carvalho Fontes, Claudio Ernani Martins Oliveira

**Affiliations:** 1 Campus Universitário Morro do Cruzeiro, Federal University of Ouro Preto (UFOP), Bauxita, Ouro Preto 35400-000, MG, Brazil; alaide.santos@aluno.ufop.edu.br (A.M.d.S.); rovadavia@ufop.edu.br (R.A.d.J.R.); wanna.fontes@ufop.edu.br (W.C.F.); 2 Campus de Itabira, Federal University of Itajubá, Rua Irmã Ivone Drummond, 200, Itabira 35903-087, MG, Brazil; vivianygm@unifei.edu.br

**Keywords:** carbon nanotubes, silica fume-assisted dispersion, low-carbon mortar

## Abstract

This study investigates the use of silica fume as a potential physical medium for carbon nanotube (CNT) incorporation in cementitious mortars, aiming to enhance mechanical performance and improve cement-use efficiency. Four mixtures were produced with a constant water-to-binder ratio of 0.50: a reference mortar (REF), a mortar incorporating 0.2 wt.% CNTs (REFCNT), a mortar with 10 wt.% cement replacement by silica fume (REFSIL), and a hybrid system containing both CNTs and silica fume (SILCNT). CNTs were introduced using a dry pre-mixing approach with silica fume, avoiding the use of surfactants, chemical functionalization, or ultrasonication. The incorporation of CNTs alone resulted in limited mechanical efficiency, leading to a reduction in flexural tensile strength at 7 days and marginal improvements at 28 days. In contrast, the hybrid SILCNT system exhibited the best overall performance, with increases of 6.2% in flexural tensile strength, 13.7% in axial compressive strength, and 16.0% in prismatic compressive strength at 28 days, indicating improved mechanical efficiency of the composite system. Regarding the environmental indicator (EPI), REFSIL and SILCNT showed a reduced value (0.68 kgCO_2_/MPa, respectively) compared to REF and REFCNT (~0.86 kgCO_2_/MPa). The results suggest that the combined use of silica fume and CNTs improves the mechanical efficiency of cementitious composites, leading to lower cement-based CO_2_ emission indicators. The role of silica fume in potentially facilitating CNT distribution is proposed as a plausible hypothesis based on indirect evidence, including mechanical performance trends and microstructural observations.

## 1. Introduction

The incorporation of nanomaterials into cementitious matrices has emerged as an effective strategy for improving the mechanical performance and durability of cement-based materials, primarily through microstructural refinement. These improvements are generally attributed to multiple mechanisms operating at the nanoscale, enabling inherently brittle and porous materials such as cementitious composites to exhibit enhanced engineering properties. Among these nanomaterials, carbon nanotubes (CNTs) have attracted particular attention, since their first characterization, owing to their exceptional tensile strength, high Young’s modulus, and multifunctional reinforcing potential [1,2,3,4,5,6,7,8,9,10,11].

Because of their high specific surface area and surface energy, nanomaterials act as preferential nucleation sites for the precipitation of cement hydration products. There are studies that discuss how carbon nanotubes can accelerate cement hydration by promoting a more rapid and homogeneous formation of calcium silicate hydrate (C–S–H) gel around the nanoparticles. This nucleation effect potentially contributes to shorter setting times and to the development of a denser C–S–H network with a higher degree of polymerization [2,3,4].

Due to their nanoscale dimensions (typically 1–100 nm), nanomaterials occupy interstitial voids and nanopores between cement grains and hydration products [1,11]. Mercury intrusion porosimetry studies have shown that the incorporation of CNTs reduces total porosity and refines the pore size distribution by converting larger capillary pores into finer pores. This pore refinement contributes to the formation of a denser and less permeable cementitious matrix [9,12,13,14,15].

Unlike conventional macro- and microfibers, which primarily restrain the propagation of existing cracks, one-dimensional nanomaterials such as CNTs and carbon nanofibers (CNFs) influence the initiation and propagation of cracks at the nanoscale [6,9,16]. Through crack-bridging mechanisms, these nanomaterials span nanopores and microcracks, facilitating stress transfer across discontinuities and dissipating fracture energy through pull-out or debonding processes [3,5,10,17].

Consequently, significant strength improvements are frequently reported. Previous investigations have observed improvements in compressive, tensile and flexural strengths, depending on the type, dosage, and dispersion quality of the nanomaterial [5,7,9,10,16,17,18]. In addition, the refined microstructure also improves durability by reducing water permeability and the ingress of aggressive ions, such as chlorides, while also mitigating autogenous and drying shrinkage [2,4,17,19].

Despite the exceptional mechanical properties of carbon nanotubes, the effective transfer of these properties to cementitious matrices depends critically on their state of dispersion and distribution within the matrix. The literature consistently recognizes that homogeneous dispersion is a fundamental prerequisite for CNTs to function as reinforcing elements rather than as sources of structural defects. CNTs exhibit a strong intrinsic tendency to form agglomerates and bundles owing to their nanoscale dimensions, high specific surface area, and strong van der Waals attractive forces. To effectively contribute to crack bridging and pore filling, individual nanotubes must be adequately separated and uniformly distributed to allow intimate contact with cement hydration products [10,19,20,21].

To overcome CNT agglomeration, several dispersion strategies have been reported. Ultrasonication is the most widely used physical method for separating CNT bundles in aqueous suspensions prior to mixing [7]. The use of surfactants, particularly polycarboxylate-based superplasticizers, represents the group of non-covalent chemical methods and improves dispersion stability through electrostatic repulsion and steric hindrance while preserving the intrinsic structure of the nanotubes [9,10,22,23]. Acid treatments are examples of covalent functionalization methods that introduce functional groups—such as carboxyl (–COOH) and hydroxyl (–OH)—onto the CNT surface, improving wettability and enhancing the interfacial interaction between the CNTs and the C–S–H gel [2,22]. Supplementary mineral materials can also be used as a physical CNT dispersion aid. The ultrafine spherical particles of silica fume (SF) can penetrate between CNT bundles during mixing, promoting mechanical separation while simultaneously densifying the matrix through pozzolanic activity [9,23,24]. Conventional dispersion methods can achieve satisfactory results but are often associated with higher costs, processing complexity, potential structural damage to CNTs, and limited scalability for construction applications. Therefore, simplified and scalable dispersion strategies remain essential for practical implementation [4,7,20,25].

On the other hand, the incorporation of poorly dispersed CNTs or excessive CNT contents compromises these benefits and can impair composite performance due to several distinct phenomena. CNT agglomerates act similarly to internal voids or weak regions within the matrix because they fail to integrate effectively with surrounding hydration products [15,19]. They also create localized stress concentrations that facilitate the initiation and propagation of microcracks under mechanical loading, reducing reinforcing efficiency [10,20]. Large agglomerates locally restrict water access to neighboring cement particles, contributing to a less homogeneous and more porous microstructure [7,15,19,20]. Poor dispersion reduces the effectiveness of pore refinement, resulting in increased porosity and permeability. This microstructural deterioration facilitates the ingress of aggressive agents (e.g., chloride ions and carbon dioxide), adversely affecting long-term durability [9,26].

### 1.1. The Role and Optimum Dosage of Silica Fume

Silica fume is widely employed in cementitious systems due to its ultrafine particles and high specific surface area, which contribute to matrix densification through filler and pozzolanic effects [14,24,27]. Several studies have identified a cement replacement level of approximately 10 wt.% SF as one of the most effective dosages for optimizing performance. When the addition of silica fume exceeds 15 wt.%, the material may cease to act predominantly through chemical (pozzolanic) effects and begin to operate primarily through physical (filler) effects, which considerably increases the water demand of the mixture [19,27,28,29].

Comparative studies explicitly evaluating silica fume in cement pastes and concrete found that mixtures containing 10 wt.% SF exhibited higher compressive strength after 90 days of curing [27]. Furthermore, the greatest reductions in total porosity are frequently observed for replacement levels up to 10 wt.%, whereas further increases in SF content often result in only marginal additional reductions. Microstructural investigations show that replacing cement with 10 wt.% SF substantially improves the interfacial transition zone, reducing its thickness [28,29].

When utilized as a physical aid for the incorporation and distribution of CNTs, previous studies have reported that a 10 wt.% SF addition significantly improves the distribution of carbon-based nanomaterials within the matrix. In one investigation, specimens containing 0.15 wt.% CNTs and 10 wt.% SF exhibited the highest compressive strength among the evaluated mixtures [7,24]. However, in most literature historical context, the contribution of silica fume has been predominantly interpreted from a pozzolanic perspective, leaving its direct role in CNT dispersion insufficiently explored.

### 1.2. Methodological Considerations

Most available studies focus primarily on mechanical performance, with limited consideration of the environmental implications associated with nanomodification. Portland cement production accounts for approximately 7–8% of global anthropogenic CO_2_ emissions due to clinker calcination and high manufacturing energy demands. Consequently, developing high-performance cementitious materials with lower carbon intensity is a strategic priority [4,7,20,25,30].

In this context, eco-efficiency assessments based on indicators relating mechanical performance to CO_2_ emissions provide an important framework for evaluating whether performance gains effectively offset the environmental burden. However, such quantitative approaches remain scarce in studies involving CNT-modified cementitious composites, particularly when alternative dispersion strategies are explored [4,7,9,31].

As highlighted in the literature, specimen geometry significantly influences internal stress distribution, scale effects, and failure mechanisms, often leading to variations in mechanical strength. Therefore, testing different geometries provides a comprehensive understanding of the material’s behavior and ensures reliable comparisons across different experimental standards and historical data [7,16,20].

Studies such as that by Parsekian et al. [32] have demonstrated that differences in specimen geometry, particularly between cylinders and prism halves, generate significant variations in compressive strength. It was observed that cylindrical specimens exhibited lower values than prismatic ones, with reductions depending on the strength range of the mortar.

A similar trend was reported by Souza et al. [33]. The authors observed that the shape of the specimens can result in variations on the order of approximately 10% to 25%, while the scale effect associated with the dimensions can induce additional differences of about 20% to 30%. These results indicate that the specimen shape directly influences stress distribution and the failure mechanism, thereby impacting the interpretation and comparison of results obtained through different experimental methodologies.

Another experimental parameter frequently discussed in the literature is the water-to-binder (w/b) ratio, investigated in studies such as those by Ramenazi et al. [9] and Judd et al. [34]. This factor exerts a direct influence on cement hydration, matrix porosity, and the dispersion of nanomaterials. In general, lower water-to-binder ratios contribute to an increase in mechanical strength due to the reduction in capillary porosity; however, they can hinder the adequate dispersion of carbon nanotubes, necessitating the adoption of more efficient dispersion techniques.

Therefore, this study advances the understanding of nano-modified cementitious materials by explicitly addressing silica fume as a physical dispersing agent for CNTs, rather than solely as a pozzolanic addition. It adopts an integrated approach, combining dispersion efficiency, mechanical performance, and environmental assessment based on a quantitative eco-efficiency indicator. By reframing the role of silica fume within this broader framework, this study contributes to bridging the gap between laboratory-scale nano-modification and practical, low-carbon applications in cementitious materials.

## 2. Materials and Methods

Multi-walled carbon nanotubes (MWCNTs) produced by chemical vapor deposition (CVD) were utilized. The synthesis was performed at 750 °C for 25 min under a continuous flow of argon (500 sccm), with ethylene (300 sccm) as the carbon source. The material exhibited an average density of 2.3 g·cm^−3^, a mean length of 45 µm, an average diameter of 30 nm, and a purity of 95%. Detailed synthesis conditions and catalyst specifications are reported elsewhere [35,36,37,38,39,40]. The MWCNTs were used in their pristine state, without post-treatment or functionalization.

Silica fume (Microsilex^®^, GCC; Grupo Cementos de Chihuahua, Chihuahua, Mexico), with a density of 2.3 g·cm^−3^ and SiO_2_ content exceeding 88%, was incorporated as a mineral admixture and will be referred to as silica hereafter. According to the manufacturer’s specifications, more than 95% of the material passes through the No. 325 sieve, indicating particle sizes smaller than 45 µm.

A high-early-strength Portland cement (CPV-ARI; CSN®, Companhia Siderúrgica Nacional, Volta Redonda, Brazil) was used as the binder. Standardized silica sand, composed of four particle size fractions (1.18, 0.60, 0.30, and 0.15 mm; Instituto de Pesquisas Tecnológicas do Estado de Sao Paulo, Sao Paulo, Brazil), was employed as the fine aggregate in equal proportions, following ISO specifications. The physical, chemical, and mechanical properties of the constituent materials are summarized in Table 1.

### 2.1. Specimen Preparation and Curing

**Figure 1:**
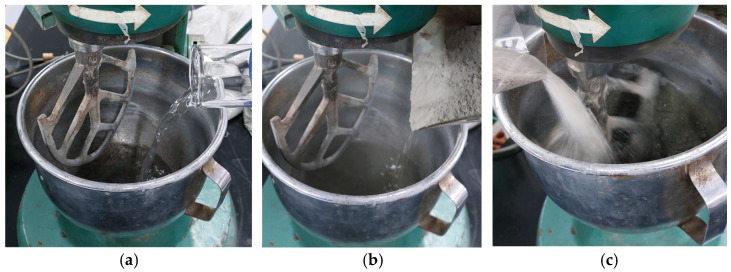
Mixing procedure: (**a**) Addition of water to the bowl with the mixer turned off; (**b**) incorporation of cement with the equipment still turned off, followed by the initiation of mixing at low speed (700 rpm) for 30 s; (**c**) gradual addition of the standardized fine aggregate under continuous agitation until complete homogenization. The total mixing time was kept constant for all formulations, ensuring the reproducibility of the process.

The production of the mortars was conducted in compliance with the procedures established in NBR 16738 [41], utilizing a vertical-shaft mortar mixer with rotation control. Initially, water was added to the bowl with the equipment turned off (Figure 1a), followed by the incorporation of cement (Figure 1b). Mixing was then initiated at a controlled low speed (700 rpm), maintaining this condition for 30 s. Next, the standardized fine aggregate was gradually added (Figure 1c), and mixing continued under continuous agitation until complete homogenization. The total mixing time (240 s) was kept constant for all formulations, ensuring the reproducibility of the procedure.

Four mortar mixtures were prepared: a reference mixture without CNTs or silica (REF); a mixture with CNTs (REFCNT); a mixture with silica (REFSIL); and a hybrid mixture (SILCNT). The reference composition (REF) was defined by a 1:3:0.5 proportion (cement: sand: water), corresponding to a water-to-cement (w/c) ratio of 0.50. For the mixtures containing silica, a constant water-to-binder (w/b) ratio of 0.50 was maintained, considering the total mass of cement, silica, and/or CNTs. The proportions of each material used in the formulated mortars are presented in Table 2.

**Figure 2:**
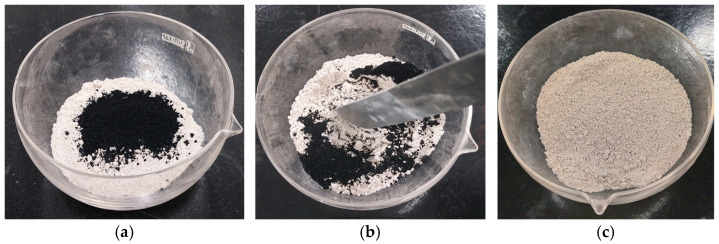
(**a**) Dry pre-dispersion procedure of carbon nanotubes (CNTs) in silica prior to incorporation into the mortar, (**b**) manual mixing of the constituents, and (**c**) production of a homogeneous material (silica + CNTs).

In REFCNT, the CNTs were incorporated at 0.2 wt.% relative to the cement mass, whereas in REFSIL, silica was used as a 10 wt.% cement replacement. In the combined system (SILCNT), silica fume replaced 10 wt.% of the cement, while CNTs were incorporated at 0.2 wt.% relative to the cement mass. In REFCNT, CNTs were previously incorporated into the dry constituents with the mixer still turned off, ensuring its initial distribution before commencing mechanical mixing. The same procedure was adopted for REFSIL, i.e., the silica fraction was added to the dry constituents before starting the mixing process, keeping the operational parameters unaltered. In SILCNT, a preliminary dry-mixing step was performed (Figure 2), in which the CNTs were manually homogenized with the silica to promote physical dispersion prior to mixing. This procedure enhances the distribution of the nanotubes by leveraging the high surface area and fine particle size of the silica. Following this step, the mortar was prepared according to the same protocol described above. No superplasticizer admixture was used.

The silica replacement level (10 wt.% of cement) was selected as a typical value within the range commonly adopted for supplementary cementitious materials (5–20 wt.%), being consistent with both the manufacturer’s recommendations and previous studies conducted by the authors’ research group [27,28,29,36,37]. The objective of this study was not to optimize the silica content, but rather to investigate its potential role as a physical aid for CNT incorporation under a typical cement replacement level. The CNT dosage (0.2 wt.% of cement) was selected based on a previous optimization study conducted by the authors, in which CNT contents of 0.10, 0.20, and 0.80 wt.% were evaluated under comparable experimental conditions. Among the investigated dosages, 0.20 wt.% exhibited the highest reinforcement efficiency, defined as the mechanical strength gain per unit mass of CNT incorporated, and was therefore adopted in the present study [38].

**Figure 3:**
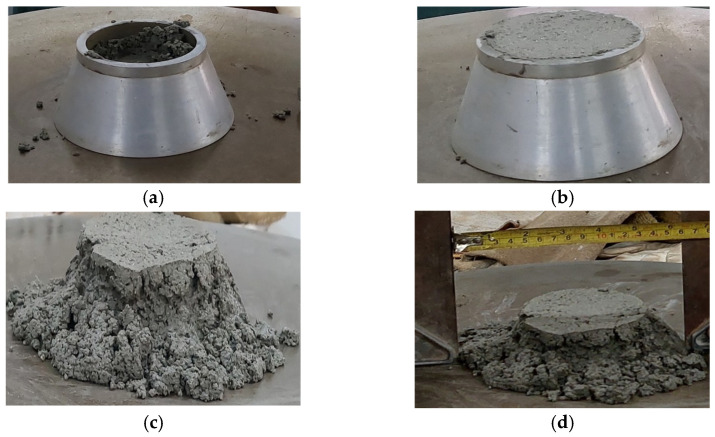
Flow table test procedure for determining mortar consistency: (**a**) Filling the mold; (**b**) struck-off surface; (**c**) flow of the mortar; (**d**) measuring the flow diameter.

Workability was assessed in accordance with NBR 13276 [42]. The test consisted of filling a truncated cone metallic mold centered on the flow table (Figure 3a). After striking off the surface, the mold was removed vertically (Figure 3b), and the table was operated, providing 30 drops within a 30 s interval (Figure 3c). The average flow diameter was determined from three orthogonal measurements (Figure 3d), with the mean value adopted as the consistency index of the mortar.

**Figure 4:**
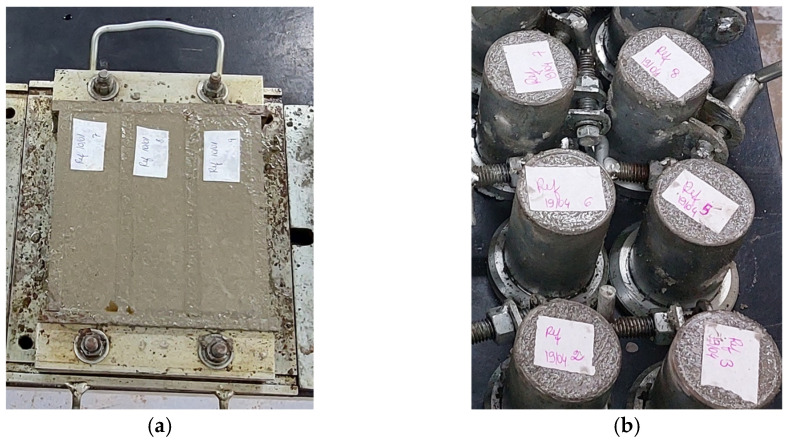
Specimen casting procedures: (**a**) Prismatic mold (40 × 40 × 160 mm); (**b**) cylindrical mold (50 × 100 mm).

After casting (Figure 4), the specimens were stored under laboratory conditions for 24 h and protected against moisture loss. Subsequently, they were demolded and subjected to water curing in a calcium hydroxide-saturated solution (limewater), maintained at room temperature (approximately 23 °C), until the designated testing ages (7 and 28 days). This curing procedure was adopted to ensure adequate hydration conditions and to minimize chemical variations in the curing environment.

**Figure 5:**
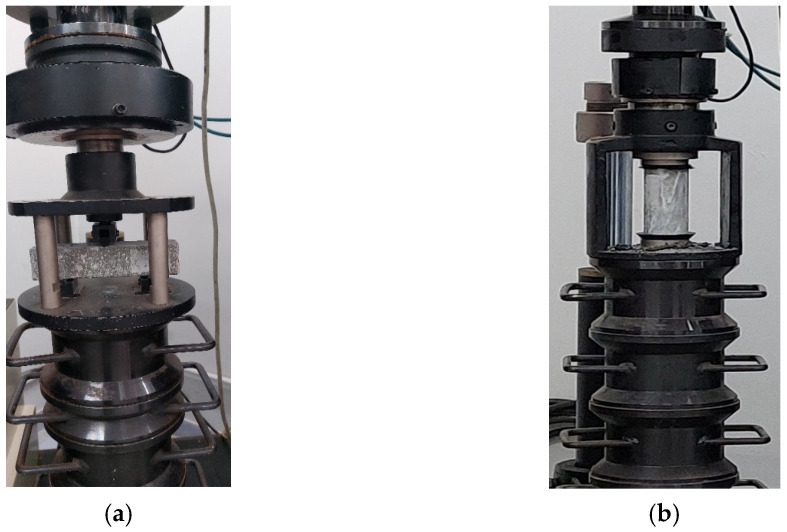
Mechanical testing procedures: (**a**) Flexural tensile strength test; (**b**) axial compressive strength test.

The determination of flexural tensile strength and compressive strength of the prismatic specimens was carried out in accordance with the Brazilian Standard NBR 13279 [43] (Figure 5a) using an EMIC DL 20000 universal testing machine (Instron Brasil Equipamentos Científicos Ltda, São José dos Pinhais, Brazil). Monotonic loading was applied at a loading rate controlled according to the requirements established by the standard. The flexural test divided each prism into two halves, resulting in a total of 36 specimens per formulation and testing age for the compressive strength assessment. The compression test on the prismatic halves was performed using the same testing machine and following the procedures specified by the standard. For simplicity, the compressive strength measured on the prismatic halves is hereafter referred to only as *compressive strength*.

For the evaluation of axial compressive strength, the cylindrical specimens were capped prior to testing to ensure plane and parallel bearing surfaces and to promote a uniform distribution of the applied load. The test was conducted in accordance with the Brazilian Standard NBR 7215 [44], under monotonic axial loading until failure (Figure 5b), while maintaining the loading rate prescribed by the standard. For each experimental condition, the reported strength values correspond to the arithmetic mean of the tested specimens.

The results were subjected to outlier elimination in accordance with standard criteria. For flexural strength and compressive strength, the limits from NBR 13279:2005 [43] were adopted, which establish the exclusion of results whose difference from the mean exceeds 0.3 MPa and 0.5 MPa, respectively. For axial compressive strength, the criterion from NBR 7215:2019 [44] was applied, which determines the elimination of values whose difference exceeds 6% of the mean.

Prior to hypothesis testing, the assumptions of normality and homogeneity of variances were assessed using the Shapiro–Wilk and Levene tests, respectively. When these assumptions were satisfied (*p* > 0.05), one-way analysis of variance (ANOVA) was performed to evaluate differences among the investigated mixtures. Whenever statistically significant differences were detected, Tukey’s honestly significant difference (HSD) post hoc test was applied for pairwise comparisons.

### 2.2. Environmental Impact Assessment

In this work, the Environmental Performance Indicator (EPI) provides a methodology to evaluate the environmental impact of cementitious materials, allowing a comparative analysis between the mechanical performance and environmental efficiency of different formulations [45,46,47]. The assessment of the environmental impact resulting from the partial replacement of cement by CNTs dispersed in silica, corresponding to a 10 wt.% reduction in the cementitious matrix, was performed using the EPI. The calculation of the EPI is based on the relationship between the CO_2_ emissions of the cementitious mixture (impact, in kg·m^−3^) and its compressive strength (MPa), as shown in Equation 1. Lower EPI values indicate greater sustainability of the mixture, as it reflects lower CO_2_ emissions per unit of mechanical performance [48]. For the calculation of CO_2_ emissions, a conversion factor of 0.9060 kgCO_2_ eq./kg^−1^ was adopted [36,48].

(1) $$EPi\left(\frac{\mathrm{Kg}{CO}_{2}}{\mathrm{MPa}}\right)=\frac{{CO}_{2}\;emissions\left(\frac{\mathrm{Kg}}{m^{3}}\right)100\;\mathrm{kg}\;solids}{compressive\;strength\;after\;28\;days}$$

## 3. Results and Discussion

### 3.1. Consistency Index

**Figure 6:**
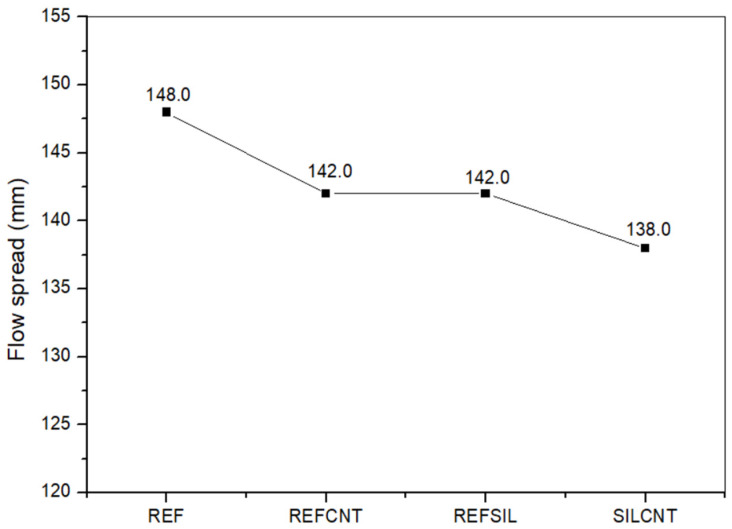
Consistency index of mortars incorporated with silica and carbon nanotubes, determined by the flow table test.

Figure 6 presents the consistency index of the evaluated mortars. The reference mixture exhibited the highest flow, whereas the modified mixtures registered reductions of 4.1% for REFCNT and REFSIL, and 6.8% for SILCNT. Therefore, a decrease in consistency was observed despite maintaining a constant water-to-binder ratio. This behavior is associated with the incorporation of high-fineness materials, whose larger specific surface area increases water demand and, consequently, reduces the plasticity of the mixture [2,5,27,28,49].

Additionally, the presence of silica and carbon nanotubes (CNTs) contributes to a decrease in the entrapped air content and the densification of the fresh matrix due to the filler effect and the physicochemical interactions promoted by micro- and nano-scale particles [49,50]. The high surface area of the CNTs enhances water adsorption on the particle surfaces, thereby restricting the mortar fluidity [4,12,13,19].

The average values obtained for REFCNT, REFSIL, and SILCNT show minor variations compared to the reference mixture, remaining close to the observed dispersion range. This behavior suggests that the low incorporation content of silica and CNTs exerted a limited influence on the consistency of the mortars.

### 3.2. Thermogravimetric Analysis

**Figure 7:**
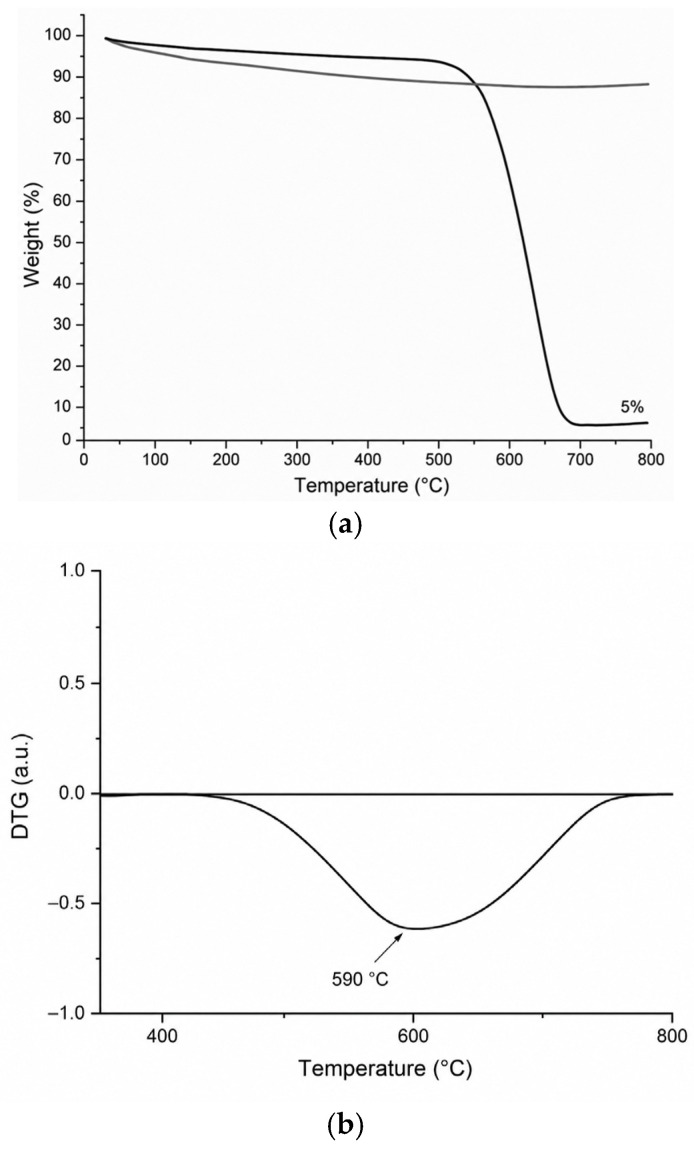
Thermogravimetric curves: (**a**) TG and (**b**) DTG of silica and carbon nanotubes.

The thermal characterization of the silica and CNTs was performed by thermogravimetry (TG) and its derivative (DTG) using a Shimadzu DTG-60 instrument (Shimadzu, Kyoto, Japan), as presented in Figure 7a,b, conducted under an oxidizing atmosphere (synthetic air). The obtained curves demonstrate distinct thermal behaviors directly related to the composition and chemical nature of each material.

The silica exhibited a mass loss below 10% up to 800 °C, mainly attributed to the removal of physically adsorbed moisture and the dehydroxylation of surface silanol groups. The absence of pronounced thermal events in the DTG curve confirms its high thermal stability, consistent with its predominantly amorphous and inorganic character [27,30].

In contrast, the CNTs displayed a sharp degradation between 550 °C and 670 °C, with a maximum mass loss rate peak at approximately 590 °C, which is characteristic of the oxidation of graphitic structures in an oxidizing atmosphere [9]. The presence of a single, well-defined peak in the DTG curve, without shoulders or secondary events at lower temperatures (≈300 °C), indicates the absence of amorphous carbon. The final residue of approximately 5% is associated with traces of metallic catalysts remaining from the synthesis process, demonstrating the high purity of the material.

Overall, the results confirm the high thermal stability of the silica and clearly delimit the thermal degradation interval of the CNTs, allowing for their unequivocal distinction within the analyzed system.

### 3.3. Mechanical Strength of Prismatic and Cylindrical Specimens

**Figure 8:**
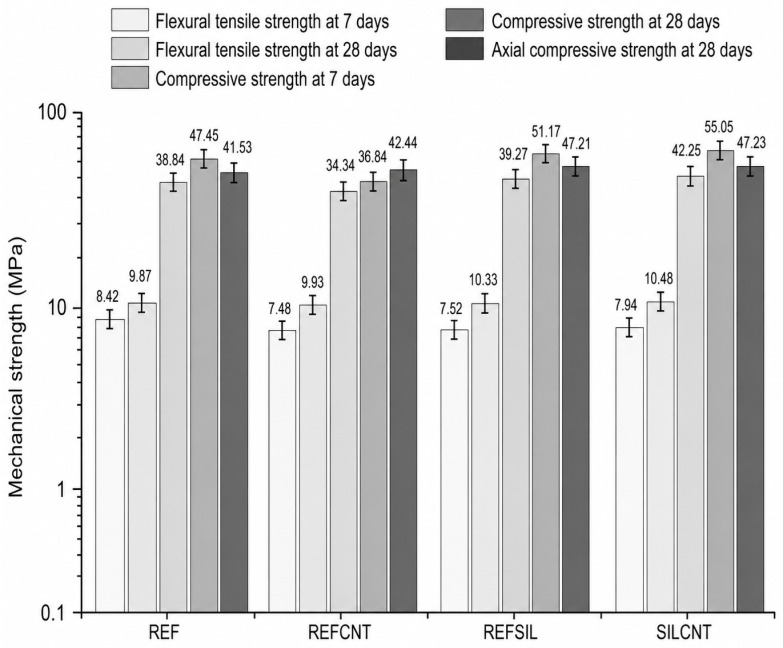
Flexural tensile strength (7 and 28 days), compressive strength (7 and 28 days), and axial compressive strength (28 days) of REF, REFCNT, REFSIL, and SILCNT mortars.

Figure 8 presents the results of the flexural tensile strength and compressive strength tests evaluated at 7 and 28 days of curing, as well as the axial compressive strength at 28 days.

Comparing the REFCNT mixture with the REF reference mixture, a reduction in mechanical performance is observed, especially in compressive strength, which might be an indication that the isolated incorporation of carbon nanotubes was not efficient under the evaluated conditions. At 28 days, REFCNT presents lower compressive and axial compressive strengths compared to REF, which suggests that the CNT dispersion may not have been adequate. The decreasing trend is also observed, albeit more moderately, in the flexural tensile strength. This behavior can be attributed to the agglomeration of the nanotubes, which, instead of acting as a reinforcement, can function as points of discontinuity within the cementitious matrix, favoring stress concentration and defect formation. Furthermore, without an efficient dispersion, the CNTs are unable to significantly promote the bridging effect, reducing their capacity to bridge microcracks and improve stress transfer [5,10,19].

At 7 days, the REFSIL and SILCNT mortars exhibited lower flexural tensile strength than the reference mortar (REF), indicating that the incorporation of silica does not immediately contributed to strength development. This behavior is consistent with the kinetics of the pozzolanic reaction, whose development is progressive and dependent on the availability of calcium hydroxide originating from cement hydration. Thus, the beneficial pozzolanic effects of silica become more pronounced at later ages, as discussed by Hong et al. [15]. Due to this evolutionary behavior, the comparative analysis was primarily conducted based on the 28-day results.

At 28 days, the isolated incorporation of either carbon nanotubes (REFCNT) or silica (REFSIL) did not promote significant variation in the flexural tensile strength compared to the reference mixture, showing differences below 0.3 MPa. In contrast, the combined formulation (SILCNT) showed an increase of 6.18%, suggesting a possible synergistic effect between the microstructural refinement promoted by the silica and the crack-bridging mechanism associated with the nanotubes.

At 28 days, the isolated incorporation of CNTs (REFCNT) resulted in a reduction of approximately 22.36% in compressive strength. This behavior may be associated with the inadequate dispersion of the nanomaterial, favoring the formation of stress concentration zones and microcracks within the cementitious matrix. Considering that the compressive strength of prism halves is determined after flexural failure, the presence of pre-existing microcracks may also contribute to greater variability in the results [16].

On the other hand, the isolated incorporation of silica (REFSIL) promoted a 7.84% increase in the compressive strength of prism halves. The SILCNT formulation showed an even more significant gain (16.02%), indicating that the simultaneous presence of silica and CNTs not only mitigates the performance loss observed in REFCNT but also enhances the strength gain compared to the isolated use of silica. This result suggests that silica acted as an auxiliary agent in the nanotube dispersion, favoring a more homogeneous distribution within the matrix.

In the cylindrical specimens at 28 days, the isolated incorporation of CNTs resulted in a variation of approximately 2% in the axial compressive strength compared to the reference, a value below the previously established relevance limit (6%). Silica promoted an increase of about 13.9%, an effect attributed to the development of the pozzolanic reaction at later ages in REFSIL and SILCNT.

Similar results were reported by Mishra [50], who attributed the strength increase to the bridging effect provided by the nanotubes, which favors stress transfer within the cementitious matrix. Consistently, the findings of this study indicate that the silica-mediated dispersion of CNTs enhances their mechanical performance, resulting in superior behavior compared to the isolated additions.

The 28-day axial compressive strength results indicate minor gains with the incorporation of CNTs, which can be explained by the operating mechanism of this nanomaterial. In the occurrence of the so-called bridging effect of CNTs, it would be more pronounced under conditions where crack opening and propagation occur, such as in flexural tensile tests, where the nanotubes would act by connecting crack faces and promoting stress transfer. In contrast, in axial compression tests, failure is predominantly governed by crushing mechanisms and the internal coalescence of microdefects, with limited crack opening. This stress state may reduce the mobilization of the bridging effect, restricting the contribution of CNTs to strength gain. Thus, the performance of the nanotubes is expected to be more discrete in compression, resulting in less significant increments compared to those observed under tensile stresses.

### 3.4. Statistical Analysis of Variability and Experimental Reliability

After applying the outlier criteria, the remaining data were used to calculate the mean, standard deviation (SD), coefficient of variation (CV), and standard error of the mean (SE), as presented in Figure 8 and Table 3. Overall, the results indicate high experimental consistency and good reliability of the obtained means.

The standard deviation values remained low for all evaluated properties, demonstrating limited dispersion around the means. This behavior is also confirmed by the coefficients of variation, which ranged between 0.45% and 4.19%, a range considered low for testing involving cementitious materials.

The low standard error values indicate good precision in the estimates of the means. For the mechanical tests, 18 specimens per mixture were initially molded; however, after applying the standard normative criteria for the elimination of outliers, the final number of samples considered in the analysis varied among the mixtures, as shown in Table 3.

In the consistency test, conducted with six measurements for each mixture, SILCNT exhibited an average flow of 138 mm, a value slightly lower than that observed for the REFCNT (142 mm) and REFSIL (142 mm) mixtures. This reduction indicates lower fluidness of the mixture, possibly associated with the increase in the specific surface area of the system containing silica and carbon nanotubes.

In the flexural tensile strength tests, the SILCNT mixture presented the highest mean among the analyzed formulations, reaching 10.48 MPa, while the REFCNT and REFSIL mixtures showed means of 9.93 MPa and 10.33 MPa, respectively. These results correspond to an approximate increase of 5.5% compared to REFCNT and 1.5% compared to REFSIL.

For the compressive strength obtained from the prismatic specimens, the SILCNT mixture presented a mean of 55.05 MPa, a value significantly higher than that observed for REFCNT (36.84 MPa), representing an increase of approximately 49%.

In the axial compression test, REFSIL and SILCNT presented the highest average strength value, reaching 47.21 and 47.23 MPa, respectively, while REFCNT showed a mean of 42.44 MPa. These results correspond to an approximate increase of 11% in mortars containing silica.

The percentage variation relative to the reference mixture (Δ vs. REF) also allows for quantifying the magnitude of the differences between the formulations. It is observed that the mixtures containing silica exhibited more significant changes when compared to the system containing only carbon nanotubes, suggesting that the observed modifications in the mechanical properties reflect consistent trends rather than just random experimental variations. In this context, the SILCNT mixture combined high strength values with low statistical variability, indicating a possible synergistic effect between the silica and the dispersed carbon nanotubes on the mechanical performance of the mortars.

In general, the combination of low SD, CV, and SE values, associated with an adequate number of samples, indicates reduced data variability and high statistical reliability, allowing a robust interpretation of the differences observed between the formulations. These results demonstrate that the experimental program was conducted with appropriate control and precision, ensuring robustness to the comparative analysis between the evaluated mixtures. The literature also highlights the importance of these indicators in evaluating experimental results in cementitious materials [51].

In a complementary manner, Huamam et al. [52], when analyzing the variability of compressive strength across different laboratories according to ASTM C39, highlighted that the coefficient of variation constitutes an important indicator of experimental precision, with values in the range of approximately 4–5% being typical for tests conducted under properly controlled laboratory conditions. Thus, the use of these statistical parameters not only allows for quantifying the variability of the results but also provides consistent support for the comparative interpretation of the performance of the evaluated mixtures.

To evaluate whether the differences among the investigated mixtures were statistically significant, one-way analysis of variance (ANOVA) was performed for the flexural tensile strength, compressive strength, axial compressive strength, and consistency index results. Prior to ANOVA, the assumptions of normality and homogeneity of variances were verified using the Shapiro–Wilk and Levene tests (Table 4), respectively. Since all datasets satisfied these assumptions (*p* > 0.05), Tukey’s honestly significant difference (HSD) test was applied for pairwise comparisons whenever significant differences were detected. Statistical significance was established at a 95% confidence level (*p* < 0.05).

The statistical analysis revealed significant differences among the investigated mixtures for all evaluated properties. For flexural tensile strength and axial compressive strength, REFSIL and SILCNT exhibited significantly higher values than REF and REFCNT, while remaining statistically equivalent to each other (*p* < 0.001). In contrast, all mixtures differed significantly in compressive strength, with SILCNT exhibiting the highest average value. For the consistency index, ANOVA also indicated significant differences (*p* = 0.011), although Tukey’s post hoc test showed that only the SILCNT mixture exhibited a significantly lower consistency than the reference mortar.

The reduced consistency observed for the SILCNT mixture did not compromise its mechanical performance. On the contrary, despite exhibiting the lowest workability, this mixture consistently achieved the highest average strength values among all investigated mortars. Since reduced workability generally hinders compaction, increases entrapped air, and tends to increase porosity, the observed improvements cannot be explained by differences in fresh-state consistency alone. Instead, the results suggest that the superior mechanical performance resulted from the combined effects of silica, including its pozzolanic activity, filler effect, matrix densification, and the proposed improvement in the physical distribution of CNTs promoted by the dry pre-mixing procedure. Therefore, workability should be regarded as a secondary factor rather than the primary mechanism responsible for the mechanical improvements observed in this study.

Overall, these findings indicate that silica played a major role in enhancing the mechanical performance of the cementitious composites. Furthermore, the statistically significant increase observed for the SILCNT mixture in prismatic compressive strength suggests a synergistic contribution arising from the combined incorporation of silica and CNTs.

The reinforcing efficiency of CNTs in cementitious composites is widely recognized to depend on their dispersion state. Numerous studies have consistently shown that improvements in mechanical performance are associated with well-dispersed CNTs, whereas insufficient dispersion promotes the formation of agglomerates that act as stress concentrators and microstructural defects, frequently resulting in limited or even detrimental mechanical performance. Therefore, the relationship between CNT dispersion and mechanical behavior is well established in the literature. Although the present results do not constitute direct evidence of CNT dispersion, the superior mechanical performance of the SILCNT mixture—together with the SEM observations, the statistical analysis, and the proposed dry pre-mixing procedure—is consistent with the hypothesis that silica promoted a more homogeneous physical distribution of CNTs within the cementitious matrix. This interpretation should be regarded as a plausible mechanism supported by the convergence of independent evidence rather than as definitive proof of improved CNT dispersion.

Notwithstanding the promising mechanical findings, it is important to acknowledge that this study focused exclusively on short-term mechanical properties and fresh-state behavior. Given that silica is widely recognized for its capacity to refine pore structures and enhance the durability of cementitious systems, evaluating properties such as water absorption, sorptivity, permeability, or chloride penetration is important to comprehensively determine the long-term performance and practical viability of the proposed SILCNT composite. Consequently, the lack of durability indicators represents a limitation of the current stage of this research.

Future studies employing direct characterization techniques, such as transmission electron microscopy (TEM), Raman mapping, or quantitative image analysis, as well as comprehensive durability testing programs, are recommended to further validate the proposed dispersion mechanism and ensure the long-term service life of these advanced composites.

### 3.5. Scanning Electron Microscopy (SEM) Analysis

**Figure 9:**
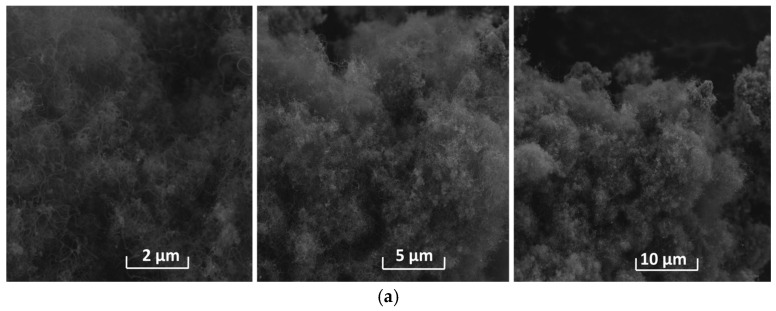

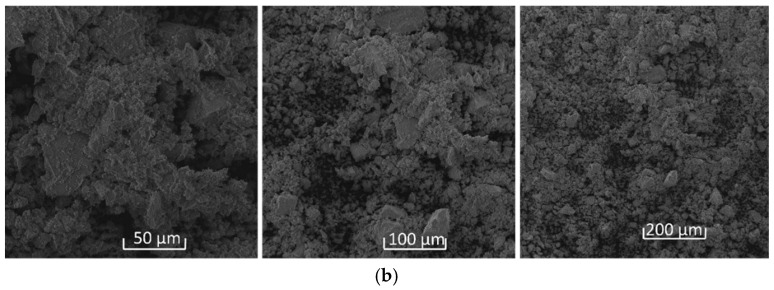
Micrographs obtained by scanning electron microscopy (SEM) of the raw materials used: (**a**) carbon nanotubes at scales of 2, 5, and 10 µm, and (**b**) silica at scales of 50, 100, and 200 µm.

Figure 9 presents micrographs obtained by scanning electron microscopy conducted with a VEGA3 TESCAN microscope (TESCAN Group, Brno, Czech Republic). They demonstrate the morphological differences between carbon nanotubes and silica. In Figure 9a, corresponding to the carbon nanotubes, a highly entangled filamentous structure is observed, forming a three-dimensional entanglement similar to a network or ‘cloud’. This morphology has also been reported by Petrov et al. [26]. As the scale increases (2 µm, 5 µm, and 10 µm), it becomes possible to identify more clearly the individual filaments of the nanotubes, which exhibit a high aspect ratio and a natural tendency to agglomerate due to van der Waals forces. This morphology results in the formation of dense and porous agglomerates, characteristic of nanotubular materials, which can hinder their homogeneous dispersion within cementitious matrices.

In Figure 9b, referring to the silica, a distinct morphology is observed, characterized by predominantly granular and agglomerated particles, forming irregular structures at larger scales. These particles present significantly smaller dimensions than typical Portland cement particles, although they appear grouped in micrometric clusters, a result of the strong surface interaction between the ultrafine silica particles. This feature contributes to the high filling effect and to its high pozzolanic reactivity in cementitious systems.

The micrographs highlight the striking structural difference between the materials: while the nanotubes exhibit a fibrous and highly entangled morphology, the silica displays a granular and agglomerated morphology, with ultrafine particles that can act in refining the matrix microstructure. This morphological complementarity is relevant when both materials are used together, as the silica can contribute to improving nanotube dispersion and particle packing within the cementitious matrix.

**Figure 10:**
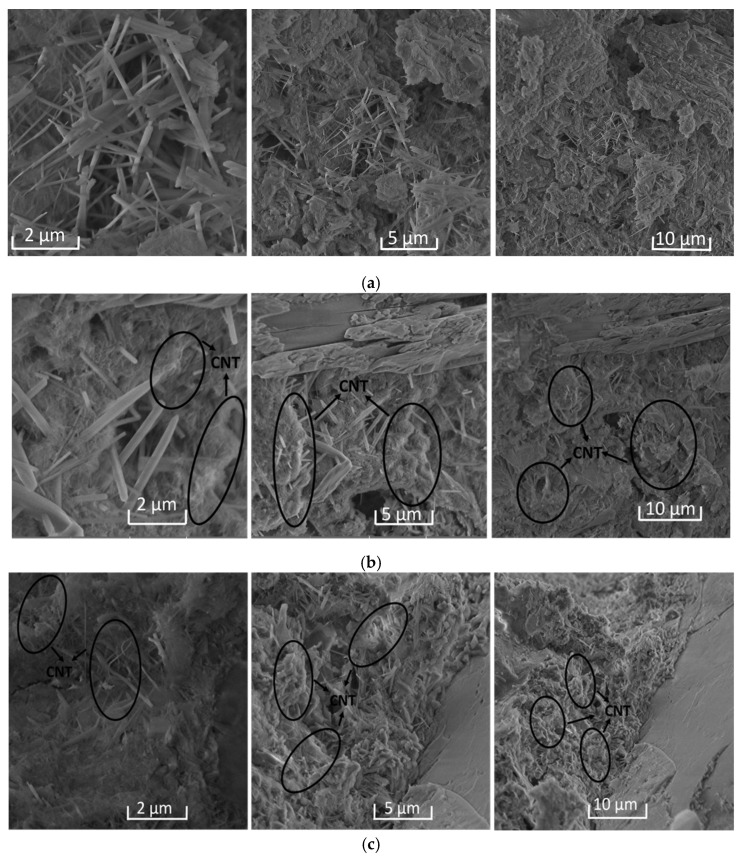
SEM micrographs of the mortars: (**a**) Silica mortar (REFSIL) after 7 days of curing; (**b**) mortar containing silica and carbon nanotubes (SILCNT) after 7 days of curing; and (**c**) SILCNT mortar after 28 days of curing.

Figure 10 presents the micrographs obtained by scanning electron microscopy (SEM) of the mortars containing silica and carbon nanotubes dispersed in silica at 7 and 28 days of curing, showing the microstructural evolution of the cementitious matrix.

In Figure 10a, the microstructure of the mortar containing silica is observed. The presence of hydration products distributed throughout the cementitious matrix stands out, featuring elongated crystals with an acicular morphology of micrometric dimensions, typically associated with ettringite formation during the early stages of cement hydration. Surrounding these structures, a denser and irregular matrix is identified, attributed to the high pozzolanic reactivity of the silica. This material reacts with the calcium hydroxide released during cement hydration, promoting the additional formation of C–S–H gel, which is primarily responsible for the mechanical strength of cementitious composites. Furthermore, due to its ultrafine particle size, silica also exerts a physical filling effect, favoring better particle packing and microstructural refinement. Consequently, a more compact matrix is observed, with a potential reduction in porosity and improvements in the mechanical and durability properties of the mortar [27,28,29].

Figure 10b presents the micrographs of the mortar containing CNTs dispersed in silica at 7 days of age. The presence of nanotube bundles located between pores and microcracks within the cementitious matrix is observed. This behavior characterizes the so-called bridging effect, in which the nanotubes connect adjacent surfaces of the matrix, contributing to the restriction of crack propagation and, hypothetically, to the increase in both flexural tensile and compressive strengths [3,5,10,17]. According to Kong et al. [24], small additions of silica, around 10%, favor the individual dispersion of CNTs among the hydration products. With an increase in the silica content, the nanotubes tend to exhibit a more uniform distribution, forming dense and relatively homogeneous regions within the cementitious matrix [25].

In the micrograph obtained at the 2 µm scale, a predominance of acicular crystals distributed throughout the hydrated matrix is observed, a typical feature of ettringite at early ages. Among these products, regions with a filamentous morphology and an entangled appearance stand out; these are associated with the CNTs, which appear partially enveloped by the hydration products. This configuration suggests a direct interaction between the nanotubes and the cementitious matrix, indicating that these nanostructures can act as nucleation sites for the formation of hydrated products, favoring local densification of the microstructure. At the intermediate scale (5 µm), CNT agglomerates with an irregular texture are identified, distributed among the hydration products. Although mechanical dispersion in silica contributes to the incorporation of the nanotubes into the matrix, a certain agglomeration tendency is still observed. However, these agglomerates remain anchored to the cementitious matrix, potentially contributing to microstructural reinforcement mechanisms such as microcrack bridging and stress transfer. At a broader scale (10 µm), the microstructure exhibits a heterogeneous character, composed of hydration products, particles associated with silica, and regions containing nanotubes partially covered by the hydrated compounds, indicating interfacial integration with the matrix.

Figure 10c presents the microstructure of the mortar containing CNTs dispersed in silica after 28 days of curing. At this age, a significantly more compact matrix is observed, reflecting the evolution of the hydration process and the progressive filling of voids by products such as C–S–H gel. Compared to the early ages, the acicular crystals become less evident, indicating a higher degree of hydration. In the highlighted regions, agglomerates with a filamentous morphology associated with the carbon nanotubes are identified, which are partially incorporated and covered by the hydrated matrix. This configuration suggests that the CNTs remain integrated into the microstructure, acting as anchoring points and contributing to the interaction with the hydration products.

At the 5 µm and 10 µm scales, regions containing nanotube agglomerates distributed along the cementitious matrix and enveloped by hydration products are observed. Even though a portion of the CNTs remains clustered, it is noted that these structures are well integrated into the matrix, indicating that the hydrated products develop around these nanostructures. This behavior favors microstructural densification and can increase the interfacial bonding between the nanotubes and the cementitious matrix [12,13,50]. With the advancement of curing age, a large part of the silica reacts with the cement, promoting the additional formation of C–S–H gel and contributing to pore blocking and microstructural refinement. Furthermore, while the hydration crystals tend to grow in a relatively rectilinear, branch-like manner, the nanotubes can be distinguished by the characteristic curvature of their fibers, which facilitates their identification in the micrographs [12,13,14,24,50].

In general, the micrographs indicate that the evolution of hydration over time promotes a progressively denser and more homogeneous microstructure. They suggest that the use of silica as a dispersion medium contributes to the distribution of the nanotubes within the cementitious matrix and favors their interaction with the hydration products. As a result, a more refined microstructure is observed, with a potential reduction in porosity and improvement in the microstructural integrity of the cementitious composite.

### 3.6. Environmental Impact Analysis

**Figure 11:**
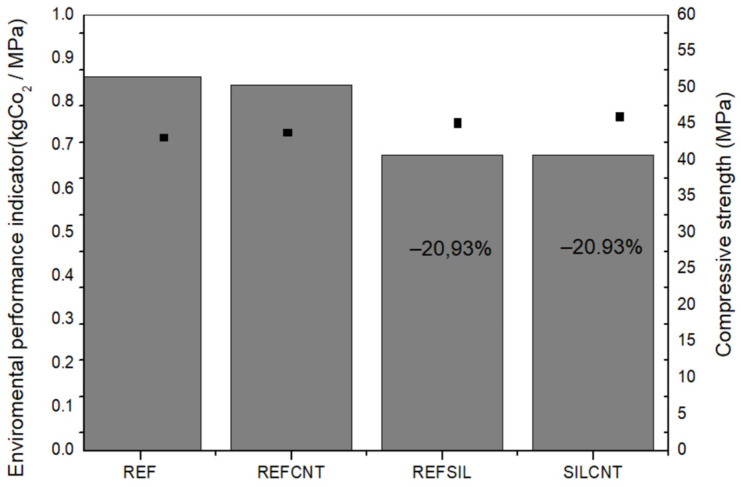
Environmental performance indicator of the REF, REFCNT, REFSIL, and SILCNT mixtures (Squared bullets representing mean compressive strength.

The lower values of the environmental performance indicator observed for the silica-containing mixtures (Figure 11), particularly SILCNT, are primarily associated with improved mechanical efficiency, which enables a reduction in cement demand to achieve equivalent or higher strength levels. Since cement production is the dominant source of CO_2_ emissions in conventional cement-based materials, this improved cement-use efficiency directly translates into lower values of the cement-based emissions indicator adopted in this study.

In this context, the SILCNT and REFSIL exhibited a 20.93% reduction in the CO_2_ emission indicator relative to the reference mixture. These results indicate that the incorporation of silica, alone or in combination with CNTs, enhances the mechanical efficiency of the cementitious system, thereby improving its performance in terms of cement-related environmental indicators.

It is important to emphasize that the environmental performance indicator used in this study is based on a simplified cradle-to-gate approach considering only cement production, which represents the dominant contribution to CO_2_ emissions in conventional cementitious systems. Therefore, the reported reductions should be interpreted strictly within this defined system boundary and do not represent a full life cycle assessment of the materials. The environmental impacts associated with carbon nanotube production, as well as other supplementary constituents, were not included in the analysis.

The observed improvement in mechanical performance of silica-containing mixtures contributes to a reduction in the amount of cement required per unit of strength, which explains the trend observed in the environmental indicator. This behavior is consistent with previous studies reporting that supplementary cementitious materials enhance the efficiency of cement-based composites by improving their mechanical performance and reducing clinker demand [47,53,54,55].

In this regard, the results highlight the potential of mixture optimization strategies based on supplementary cementitious materials to improve cement-use efficiency, which is widely recognized as a key pathway for reducing the environmental impact of cement-based systems. However, a comprehensive life cycle assessment including CNT production, silica processing, and all upstream processes is necessary for a complete evaluation of environmental performance and is recommended for future work.

## 4. Conclusions

The proposed dry pre-mixing strategy combining silica and carbon nanotubes (CNTs) was shown to be a promising and technically feasible approach for producing high-performance cementitious mortars without the use of surfactants, chemical functionalization, or energy-intensive dispersion techniques such as ultrasonication. Among the investigated mixtures, the hybrid system (SILCNT) exhibited the best overall mechanical performance after 28 days, reaching average strengths of 10.48 MPa in flexural tension, 55.05 MPa in prismatic compression, and 47.23 MPa in cylindrical compression. Compared with the reference mixture, these values correspond to increases of 6.18%, 16.02%, and 13.70%, respectively.

The statistical analysis confirmed that silica played a major role in improving the mechanical performance of the investigated composites. The hybrid mixture consistently exhibited equal or superior average performance in all mechanical tests (except for flexural tensile strength after 7 days). Furthermore, despite presenting the lowest consistency index, the SILCNT mixture achieved the highest mechanical strengths. Since reduced workability generally impairs compaction and tends to increase porosity, these results indicate that the observed strength enhancement cannot be attributed to differences in fresh-state workability alone, but rather to the combined effects of silica and CNT incorporation.

Scanning electron microscopy revealed a denser microstructure in the silica-containing mixtures and suggested good integration of CNTs within the hydrated cementitious matrix. Although direct characterization of CNT dispersion was beyond the scope of this study, the convergence of the mechanical performance, statistical analysis, SEM observations, and the well-established relationship between CNT dispersion and mechanical behavior reported in the literature supports the hypothesis that the proposed dry pre-mixing procedure promoted a more homogeneous physical distribution of CNTs within the cementitious matrix. Accordingly, the role of silica as a physical dispersion aid should be regarded as a plausible mechanism supported by indirect evidence rather than as a definitive demonstration.

From an environmental perspective, the proposed methodology also offers potential sustainability benefits. Considering the system boundaries adopted in this study, the partial replacement of cement by silica and the optimized use of CNTs resulted in an estimated 20.93% reduction in cement-related CO_2_ emissions. It should be emphasized, however, that this indicator represents a simplified cradle-to-gate assessment based solely on cement consumption and does not constitute a complete life cycle assessment. Consequently, the environmental impacts associated with CNT production, silica processing, energy consumption, and other upstream processes were not included and should be addressed in future studies.

Overall, the proposed dry pre-mixing strategy represents a simple, low-cost, and potentially scalable route for incorporating CNTs into cementitious materials while avoiding conventional dispersion procedures based on surfactants, chemical functionalization, or ultrasonication. Although further investigations employing direct characterization techniques—such as transmission electron microscopy (TEM), Raman mapping, or quantitative image analysis—are still required to conclusively validate the proposed dispersion mechanism, the results demonstrate the considerable potential of this methodology for producing high-performance cementitious composites through a process that is compatible with practical and large-scale industrial applications.

## 5. Patents

BR 10 2023 011891 7, título: “PROCESSO DE OBTENÇÃO DE CIMENTO NANOESTRUTURADO USANDO SÍLICA COMO AGENTE DISPERSOR NATURAL DE NANOTUBOS DE CARBONO COMO FEITOS”, Morais; V.G; Morais, E.A; Oliveira, C.E.M.; Silva, E.E.; Ribas, R.A.J.; Santos, A.M.; Brasil. INPI—Instituto Nacional da Propriedade Industrial. Deposit: 15 June 2023.

## Figures and Tables

**Table 1:** Physical, chemical, and mechanical properties of the constituent materials used in mortars.

CPV-ARI Cement		
Property	Value/Requirement	Unit
Fineness	45 µm (≤6.0)	µm
Density	2.3	g/cm^3^
Initial setting time	≥60	min
Compressive strength—7 days	≥34	MPa
Compressive strength—28 days	-	MPa
Silica Fume		
Component	Value	Unit
SiO_2_	92.35 (≥85.0)	%
K_2_O	0.94	%
Fe_2_O_3_	0.05	%
CaO	0.19	%
Al_2_O_3_	2.21	%
SO_3_	1.52	%
ZnO	Traces	%
MnO	Traces	%
CuO	Traces	%
Rb_2_O	Traces	%
Property	Value	Unit
Modified Chapelle	1691	mg/g Ca (OH)_2_
Specific surface area	20.238	m^2^/g
Loss on ignition	2.70	%
Density	2.3	g/cm^3^
Average diameter	45	µm
Carbon Nanotubes		
Property	Value	Unit
Density	2.3	g/cm^3^
Average length	45	µm
Average diameter	30	nm
Purity	>	95%

**Table 2:** Composition of cementitious mortar formulations.

Components	Sample Mortars, in Mass			
	REF	REFCNT	REFSIL	SILCNT
Water (g)	197.5	197.5	197.5	197.5
Cement Portland(g)	395.0	394.21	355.5	354.71
Active Silica (g)	-	-	39.5	39.5
CNT (g)	-	0.79	-	0.79
Sand 16 mesh (g)	296.0	296.0	296.0	296.0
Sand 30 mesh (g)	296.0	296.0	296.0	296.0
Sand 50 mesh (g)	296.0	296.0	296.0	296.0
Sand 100 mesh (g)	296.0	296.0	296.0	296.0

**Table 3:** Statistical analysis of mortar properties in the fresh and hardened states (28 days).

Property	Mixture	Mean	SD	CV (%)	SE	Δ vs. REF (%)	*n*
Consistency (mm)	REF	148 (a)	6.2	4.19	2.53	—	6
	REFCNT	142 (ab)	4.1	2.89	1.67	−4.05	6
	REFSIL	142 (ab)	5.0	3.52	2.04	−4.05	6
	SILCNT	138 (b)	2.9	2.10	1.18	−6.76	6
Flexural tensile strength (MPa)	REF	9.87 (b)	0.16	1.62	0.05	—	10
	REFCNT	9.93 (b)	0.19	1.91	0.08	+0.61	6
	REFSIL	10.33 (a)	0.19	1.84	0.07	+4.66	8
	SILCNT	10.48 (a)	0.21	2.00	0.06	+6.18	12
Compressive strength (MPa)	REF	47.45 (d)	0.37	0.78	0.11	—	12
	REFCNT	36.84 (c)	0.36	0.98	0.12	−22.36	9
	REFSIL	51.17 (b)	0.26	0.45	0.08	+20.48	11
	SILCNT	55.05 (a)	0.28	0.51	0.09	+16.02	9
Axial compressive strength (MPa)	REF	41.53 (b)	0.91	2.19	0.26	—	12
	REFCNT	42.44 (b)	1.41	3.32	0.58	+2.19	6
	REFSIL	47.21 (a)	1.80	3.86	0.52	+12.42	12
	SILCNT	47.23 (a)	1.84	3.90	0.58	+13.73	10

Note: Mean = average value; SD = standard deviation; CV = coefficient of variation; SE = standard error; Δ = variation relative to the reference mixture (REF); *n* = number of specimens/measuments. Means followed by the same letter are not significantly different according to Tukey’s HSD test (*p* > 0.05).

**Table 4:** Statistical comparison of fresh and hardened mortar properties at 28 days.

Property	Shapiro–Wilk	ANOVA (F)	*p*-Value	Tukey HSD
Flexural tensile strength	*p* > 0.05	23.65	<0.001	REFSIL = SILCNT > REF = REFCNT
Compressive strength	*p* > 0.05	4826.39	<0.001	SILCNT > REFSIL > REF > REFCNT
Axial compressive strength	*p* > 0.05	45.90	<0.001	SILCNT = REFSIL > REFCNT = REF
Consistency index	*p* > 0.05	4.85	0.011	REF > SILCNT

## Data Availability

The original contributions presented in this study are included in the article. Further inquiries can be directed to the corresponding author.

## Abbreviations

The following abbreviations are used in this manuscript:

CNT	Carbon nanotubes
REF	Reference
REFCNT	Reference with Carbon nanotubes
REFSIL	Reference with Silica
SILCNT	Silica with Carbon nanotubes
EPI	Environmental Performance Indicator
CSH	Calcium silicate hydrate
CNF	carbon nanofibers
SF	Silica Fume
MWCNT	Multi-walled carbon nanotubes
CVD	Chemical Vapor Deposition
w/c	Water-to-cement
w/b	Water-to-binder
NBR	Brazilian Standard
TG	Thermogravimetric analysis
DTG	Derivative Thermogravimetric analysis
SD	Standard deviation
CV	Coefficient of variation
SE	Standard error
TEM	Transmission electron microscopy
SEM	Scanning electron microscopy
ABNT	Associação Brasileira de Normas Técnicas
CAPES	Coordenação de Aperfeiçoamento de Pessoal de Nível Superior
PROPPI/UFOP	Pro-Reitoria de Pesquisa e Inovação da Universidade Federal de Ouro Preto
PROPEC	Programa de Pós-Graduação em Engenharia Civil
LMCC	Laboratório de Materiais de Construção Civil
LNA	Laboratório de Nanotecnologia
UFOP	Federal University of Ouro Preto
